# Ischemia reperfusion dysfunction changes model-estimated kinetics of myofilament interaction due to inotropic drugs in isolated hearts

**DOI:** 10.1186/1475-925X-5-16

**Published:** 2006-03-02

**Authors:** Samhita S Rhodes, Amadou KS Camara, Kristina M Ropella, Said H Audi, Matthias L Riess, Paul S Pagel, David F Stowe

**Affiliations:** 1 Department of Anesthesiology, Medical College of Wisconsin, 8701 Watertown Plank Road, Milwaukee, WI 53226, USA; 2 Department of Physiology, Medical College of Wisconsin, 8701 Watertown Plank Road, Milwaukee, WI 53226, USA; 3 Cardiovascular Research Center, Medical College of Wisconsin, 8701 Watertown Plank Road, Milwaukee, WI 53226, USA; 4 Department of Pulmonary Medicine and Critical Care, Medical College of Wisconsin, 8701 Watertown Plank Road, Milwaukee, WI 53226, USA; 5 Department of Biomedical Engineering, Marquette University, 1515 W Wisconsin Ave, Milwaukee, WI 53233, USA; 6 VA Medical Center, Milwaukee, WI 53295, USA

## Abstract

**Background:**

The phase-space relationship between simultaneously measured myoplasmic [Ca^2+^] and isovolumetric left ventricular pressure (LVP) in guinea pig intact hearts is altered by ischemic and inotropic interventions. Our objective was to mathematically model this phase-space relationship between [Ca^2+^] and LVP with a focus on the changes in cross-bridge kinetics and myofilament Ca^2+ ^sensitivity responsible for alterations in Ca^2+^-contraction coupling due to inotropic drugs in the presence and absence of ischemia reperfusion (IR) injury.

**Methods:**

We used a four state computational model to predict LVP using experimentally measured, averaged myoplasmic [Ca^2+^] transients from unpaced, isolated guinea pig hearts as the model input. Values of model parameters were estimated by minimizing the error between experimentally measured LVP and model-predicted LVP.

**Results:**

We found that IR injury resulted in reduced myofilament Ca^2+ ^sensitivity, and decreased cross-bridge association and dissociation rates. Dopamine (8 μM) reduced myofilament Ca^2+ ^sensitivity before, but enhanced it after ischemia while improving cross-bridge kinetics before and after IR injury. Dobutamine (4 μM) reduced myofilament Ca^2+ ^sensitivity while improving cross-bridge kinetics before and after ischemia. Digoxin (1 μM) increased myofilament Ca^2+ ^sensitivity and cross-bridge kinetics after but not before ischemia. Levosimendan (1 μM) enhanced myofilament Ca^2+ ^affinity and cross-bridge kinetics only after ischemia.

**Conclusion:**

Estimated model parameters reveal mechanistic changes in Ca^2+^-contraction coupling due to IR injury, specifically the inefficient utilization of Ca^2+ ^for contractile function with diastolic contracture (increase in resting diastolic LVP). The model parameters also reveal drug-induced improvements in Ca^2+^-contraction coupling before and after IR injury.

## Introduction

**Figure 1:**
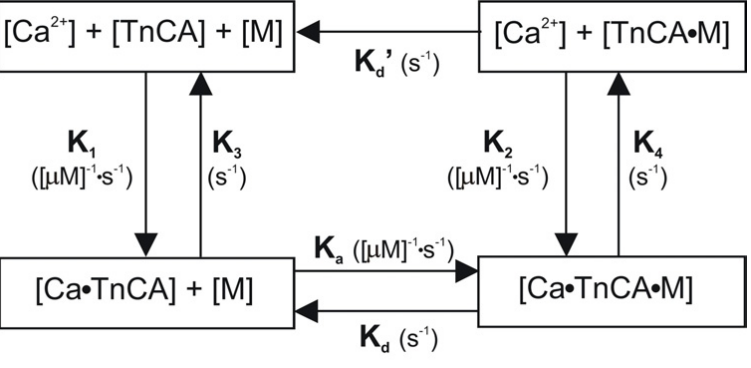
Block diagram of a biochemical model relating the input/output relationship between myoplasmic [Ca^2+^] and LVP adapted from Baran et al.[7] and Shimizu et al.[8] The 4-state model is governed by 5 differential equations. TnCA represents the troponin C molecule on the actin (A) myofilament, M represents the myosin head, + indicates weak bonds and • represents strong bonds. The sequence of events from phasic [Ca^2+^] to contraction are as follows: Ca^2+ ^binds to TnCA, tropomyosin shifts so M and A can bind forming an actinomyosin cross-bridge, Ca^2+ ^dissociates from TnCA with cross-bridge attached, and finally the cross-bridge breaks. Model rate constants and their units are indicated by their sites of action and described in Table 1. Note that A and M cannot form cross-bridges in the absence of Ca^2+^; however, since this is a loose coupling model, once a cross-bridge has been formed it no longer requires associated Ca^2+ ^to remain attached.

We have described the utility of phase-space representations of simultaneously measured myoplasmic Ca^2+ ^concentration ([Ca^2+^]) and left ventricular pressure (LVP) in guinea pig intact hearts during spharmacologic and pathophysiologic interventions [1-5]. We used several novel indices to describe the morphological changes displayed in the LVP- [Ca^2+^] relationship that occur during changes in the contractile state [3]. In the present study we extended our phase-space analyses of LVP and [Ca^2+^] [5] using mathematical modeling techniques to examine the effects of ischemia reperfusion (IR) injury on changes elicited by different positive inotropic agents. We used a theoretical four-state computational model (Figure 1, Table 1) [4,6-8] to explore mechanisms underlying formation of LVP-Ca^2+ ^loops, specifically the kinetic interactions between actin and myosin cross-bridges, and between Ca^2+ ^and troponin C with pharmacologic interventions. In a previous study we demonstrated that this four-state model is capable of reproducing the dominant characteristics of the mechanisms underlying Ca^2+^-contraction coupling during hypothermic perfusion and after normothermic short-term and hypothermic long-term IR injury [4].

**Table 1:** Model parameters and brief description.

Parameter Name	Description
K_1 _(1/μM•s)	Cooperative binding rate constant of Ca^2+ ^to TnCA
K_a _(1/μM•s)	Cooperative rate constant of formation of A•M
K_2 _(1/μM•s)	Association rate constant of Ca^2+ ^to A•M
K_3 _(1/s)	Dissociation rate constant of Ca^2+ ^from TnCA
K_4 _(1/s)	Dissociation rate constant of Ca^2+ ^from A•M
K_d _(1/s)	Dissociation rate constant of A•M in the presence of attached Ca^2+^
K_d_' (1/s)	Dissociation rate constant of A•M in the absence of attached Ca^2+^

K = rate constants characterizing the 4-state model are pictured in Figure 1 and described in detail in the Appendix. A = Actin, M = Myosin, TnCA = Troponin C on the A filament, A•M = actinomyosin cross-bridges.

Our objective was to determine how positive inotropic drugs with differing pharmacological mechanisms of action affect estimated rates of cross-bridge cycling and myofilament Ca^2+ ^handling before and after IR injury from the phase-space relationship of Ca^2+^-contraction coupling. The model parameters were estimated using data obtained from guinea pig spontaneously beating, isolated hearts at physiologic temperature)[5]. Three pharmacological classes of positive inotropes were examined: dopaminergic and adrenergic agonists (i.e., dopamine, dobutamine), a Na^+^/K^+ ^ATPase inhibitor (i.e., digoxin), and a so-called myofilament Ca^2+ ^sensitizer with phosphodiesterase inhibiting properties (i.e., levosimendan). While each of these drugs increases myoplasmic [Ca^2+^] ("upstream" mechanism), their effects on Ca^2+ ^binding to troponin C ("central" mechanism) and actinomyosin cross-bridge cycling ("downstream" mechanism) remain unexplored or controversial.

Several investigators have reported an increase in cross-bridge formation by post-synaptic adrenoceptor agonists [9,10], but others have failed to confirm these findings [11]. Digoxin may regulate formation of cross-bridges [12] but its effect on Ca^2+ ^affinity for troponin C is unclear. However, another Na^+^/K^+ ^ATPase inhibitor, ouabain, was shown to increase cross-bridge kinetics and myofilament Ca^2+ ^cycling in ventricular myocardium from patients with heart failure [13]. Ca^2+ ^sensitizers are believed to improve myofilament Ca^2+ ^sensitivity by enhancing troponin C sensitivity for Ca^2+ ^and may also alter cross-bridge attachment and detachment rate constants [14]. One of these, levosimendan, is reported to bind to troponin C in the presence of Ca^2+ ^and to stabilize the Ca^2+^- troponin C complex without increasing Ca^2+ ^binding affinity with troponin C [15]. In guinea pig papillary muscles paced at room temperature, levosimendan prolonged the attachment of cross-bridges [16], accelerated cross-bridge association and decelerated cross-bridge dissociation rate constants [17]. It is unknown if IR injury alters these effects of levosimendan on contraction kinetics. Our model provides additional insight into myofibrillar protein interactions responsible for translating phasic changes in [Ca^2+^] into myocardial contraction and relaxation before and after IR injury in the presence and absence of these inotropic drugs.

## Methods

The investigation conformed to the *Guide for the Care and Use of Laboratory Animals *from the US National Institutes of Health (NIH No. 85–23, Revised 1996). Prior approval was obtained from the Medical College of Wisconsin and Marquette University Animal Studies Committees. Guinea pig heart isolation and our fluorescence technique to measure myoplasmic free [Ca^2+^] has been detailed in previously published work [1-5,18-24], and are only briefly described here. Albino English short-haired guinea pigs (n = 40) were anesthetized with 30 mg ketamine i.p. and treated with heparin (1000 units). The animals were then decapitated when unresponsive to noxious stimulation. After thoracotomy the inferior and superior venae cavae were cut away and the aorta was cannulated distal to the aortic valve. Each heart was immediately perfused via the aortic root with a cold oxygenated, modified Krebs-Ringer's (KR) solution (equilibrated with 97% O_2 _and 3% CO_2_) at an aortic root perfusion pressure of 55 mmHg and was then rapidly excised. The KR perfusate (pH 7.39 ± 0.01, pO_2 _620 ± 10 mmHg) was filtered (5 μm pore size) in-line and has the following calculated composition in mM (non-ionized): Na^+ ^137, K^+ ^5, Mg^2+ ^1.2, Ca^2+ ^1.25, Cl^- ^134, HCO_3 _^- ^15.5, H_2_PO_4 _^- ^1.2, glucose 11.5, pyruvate 2, mannitol 16, EDTA 0.05, probenecid 0.1, and insulin 5 (U/L). Perfusate and bath temperatures were maintained at 37.2 ± 0.1°C using a thermostatically controlled water circulator. KR with reduced [CaCl_2_] (1.25 mM) allowed a wider range of inotropic responses at a lower control LVP.

LVP was measured isovolumetrically with a transducer connected to a thin, saline-filled latex balloon inserted into the LV through the mitral valve from an incision in the left atrium. Balloon volume was adjusted initially to a diastolic LVP of zero mmHg so that any subsequent increase in diastolic LVP reflected an increase in LV wall stiffness i.e., diastolic contracture. Pairs of bipolar electrodes were placed in the right atrial appendage, right ventricular apex and LV base to monitor spontaneous heart rate and atrial-ventricular conduction time. Coronary flow (aortic inflow, CF) was measured at constant temperature and perfusion pressure (55 mmHg) by an ultrasonic flowmeter (Transonic T106X, Ithaca, NY) placed directly into the aortic inflow line.

The Ca^2+ ^indicator indo-1 AM (Sigma Chemical, St. Louis, MO) was dissolved in vehicle solution consisting of 1 mL of dimethyl sulfoxide (DMSO) containing 16 % (w/v) Pluronic I-127 (Sigma Chemical) and diluted to 165 mL with modified KR solution. Each heart was then loaded with indo-1 AM for 30 min with the re-circulated KR solution, which had a final indo-1 AM concentration of 6 μM. Loading was stopped when the fluorescence (F) intensity at 385 nm increased by about 10 fold. Residual interstitial indo-1 AM was washed out by perfusing the heart with KR for another 20 min. Probenecid (100 μM) was present in the perfusate to retard cell leakage of indo-1. Fluorescence emissions at 385 and 456 nm (F_385 _and F_456_) were recorded using a modified luminescence spectrophotometer (SLM Aminco-Bowman II, Spectronic Instruments, Urbana IL). The LV region of the heart was excited with light from a xenon arc lamp and the light was filtered through a 360 nm monochromator with a bandwidth of 16 nm. Although both F_385 _and F_456 _decline over time, time control studies showed that the F_385_/F_456 _ratio remained stable indicating that the effective measured [Ca^2+^] was unchanged [19]. The total exposure time to the 350 nm excitation wavelength light was 62.5 s. Customized software was developed in MATLAB^® ^for off-line signal processing of the recorded data. The F_385_/F_456 _ratio was converted to [Ca^2+^] after correcting for background autofluorescence and non-cytosolic fluorescence as described in our previously published work. [1-5,18-24].

### Experimental protocol and data collection

Unpaced hearts were randomly assigned to receive dobutamine (4 μM), dopamine (8 μM), digoxin (1 μM), or levosimendan (1 μM) (n = 8 in each group). Each inotropic drug was infused for 2 min 30 min before global ischemia. Next, hearts were subjected to a 30 min period of global ischemia and inotropic drugs were again infused for 2 min after 30 min reperfusion. The concentrations of inotropic drugs were the approximate ED_50 _concentrations as previously established [2,3]. Eight control hearts were not exposed to inotropic interventions before or after global ischemia. At the end of each experiment MnCl_2 _(100 μM) was infused for 10 min to quench the myoplasmic indo-1 Ca^2+ ^signal to correct for the non-myoplasmic fraction. All analog signals were digitized and recorded at 125 Hz for later analysis using MATLAB^® ^as previously described [3].

**Figure 2:**
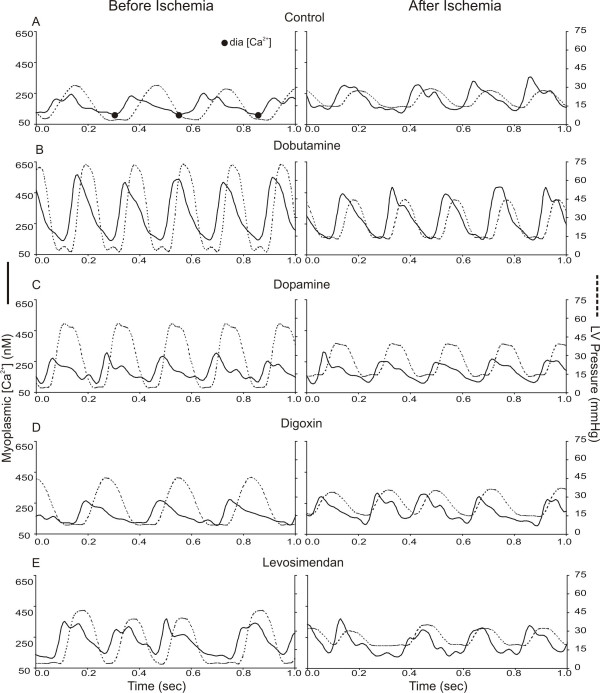
Sample time series plots of LVP and [Ca^2+^] from Control (A), Dobutamine (B), Dopamine (C), Digoxin (D), and Levosimendan (E) before (left panel) and after (right panel) ischemia [5]. Diastolic Ca^2+ ^detection points are shown for control. Extracellular CaCl_2 _was half-normal (1.2 mM) to allow for a full range of responses to the drugs. This accounts for the lower control values of systolic LVP and [Ca^2+^]. Phasic LVP was lower after ischemia in all groups but phasic [Ca^2+^] was higher only in the control group (see table 1).

Simultaneous recordings of [Ca^2+^] and LVP were obtained at selected intervals before and during administration of inotropic drugs and IR (Figure 2A–E). The timing of peak diastolic [Ca^2+^] for each cardiac cycle was obtained over the 2.5 s recordings using a simple event detection algorithm. The [Ca^2+^], and simultaneously obtained LVP signals between each consecutively detected Ca^2+ ^diastolic point, were aligned and averaged on a point by point basis to form the averaged [Ca^2+^] and LVP transient signals. Any dysrhythmic beats were excluded from the averaged [Ca^2+^] and LVP transient signals. LVP was plotted as a function of myoplasmic [Ca^2+^] over a representative cardiac cycle to create phase-space diagrams. These diagrams represent the dynamic relationship between trigger Ca^2+ ^and the resulting pressure development due to central and downstream mechanisms. Because we are unable to directly measure the interactions between Ca^2+ ^and troponin C, or the kinetics of cross-bridge cycling, we utilized a previously developed a mathematical model to help elucidate effects of changes in myofilament interaction that contribute to changing the dynamic relationship between LVP and Ca^2+^.

### Description and assumptions of mathematical model

The mathematical model utilized for the interpretation of our kinetic data allows for the interaction between troponin C attached to the actin (A) myofilament (TnCA) and myosin (M) for cross-bridge formation and force development in the presence of Ca^2+ ^[4,6-8,25]. The model (Figure 1, Table 1) consists of four stages governed by the following five differential equations:



LVP, as predicted by the model (LVP_Mod_), is proportional to the number of cross-bridges formed: [Ca•TnCA•M] + [TnCA•M] [7]. Cooperativity, the positive feedback mechanism responsible for a rise in force, has been attributed to effects of cross-bridge formation on neighboring cross-bridges and/or effects of Ca^2+ ^binding to TnCA on neighboring tropomyosin units [26,27]. In accordance with Baran et al. [7] and Shimizu et al. [8], we accounted for cooperative contraction and relaxation by allowing K_1 _and K_a _to vary according to functions described as:



The α parameter represents the slopes of the K_1 _and K_a _curves and is a measure of sensitivity of the cooperative mechanism; an increase in α represents accelerating interactions between TnCA and Ca^2+ ^(α_1_) and actinomyosin cross-bridges (α_a_). The β parameter represents the static value of K_1 _and K_a _at 0 LVP; an increase in β indicates an increase in the resting value of the affinity of TnCA and Ca^2+ ^(β_1_) and cross-bridge attachment (β_a_).

We assumed a "loose coupling" model where Ca^2+ ^dissociates from TnCA before the M head detaches from the A molecule [28]. We also assumed that the transition between weak and strong cross-bridge conformations is rapid and not rate-limiting [29]. Finally, we assumed that changes in sarcomere length had little effect on the relationship between myoplasmic Ca^2+ ^and isovolumic LVP. Kentish and Wrzosek [30] reported that lengthening of rat isolated myocardium increased twitch force but had no effect on the magnitude of the Ca^2+ ^transient, suggesting an increase in myofilament Ca^2+ ^sensitivity. In contrast, Shimizu et al. [8] reported no length-dependent alterations in myofilament Ca^2+ ^binding or cross-bridge cycling in canine isolated, blood-perfused hearts.

### Kinetic analysis

The experimentally measured and averaged Ca^2+ ^transients were lowpass filtered at 25 Hz and upsampled to 2500 Hz using a linear interpolation scheme prior to their use as model forcing functions. The governing differential equations were solved numerically using a 4^th ^order Runge-Kutta algorithm (on a MATLAB^® ^platform) with a 0.4 ms step size equal to the post-interpolation sampling interval and the following initial conditions [7,8,25,26]: [TnCA]_(t = 0) _= 70 μM, [M]_(t = 0) _= 20 μM, [Ca•TnCA]_(t = 0) _= 0 μM, [Ca•TnCA•M]_(t = 0) _= 0 μM, and [TnCA•M]_(t = 0) _= 0 μM.

Model rate constants were optimized using commercially available algorithms based on constrained quasi-Newton methods that guarantee linear convergence, and were estimated to minimize the root mean square (RMS) error between LVP_Mod _and LVP at the sampled time points.



Lower and upper bounds for optimization of 1^st ^order rate constants were set at 0 and 2000 /sec (0.5 ms step size) respectively. Initial values for the parameters were obtained from Baran et al. [7]. Several constraints were imposed on the model rate constants during optimization. To ensure a positive feedback, α_1 _and α_a _must be greater than 0. K_d_' must be greater than K_d _since cross-bridges dissociate more readily in the absence of attached Ca^2+^; K_d_' accounts for the physiological difference between contraction and relaxation kinetics [7]. The maximum rate constant of Ca^2+ ^binding to TnCA with attached cross-bridges must be greater than the maximum rate constant of Ca^2+ ^binding to TnCA with no attached cross-bridges (K_2_>K_1_); this concept incorporates the idea of a positive feedback mechanism to explain the delay in rise in LVP during contraction [31].

### Statistical analysis

Model rate constants computed before and during administration of inotropic drugs and IR were compared using one-way ANOVA followed by Dunnett's comparison of means post-hoc test (MINITAB™ Statistical Software Release 13.3, Minitab Inc, State College, PA). Post-ischemic values of control and inotropic drugs were compared to their respective pre-ischemic values using Student's paired t-test. Differences among means were considered statistically significant at *P *< 0.05 (two-tailed). All experimental measurements and model rate constants were expressed as means ± SE.

## Results

The effects of drugs and ischemia on heart rate and phasic LVP and [Ca^2+^] have been previously reported [5] and are presented again in table 2. Note that IR injury had no effect on heart rate either in the presence or absence of drugs. Conversely, dobutamine and dopamine both increased heart rate from control before and after ischemia. Digoxin did not change heart rate before or after ischemia and levosimendan increased heart rate before, but not after ischemia.

**Table 2:** Effects of inotropic drugs before and after global ischemia on morphological and temporal indices [5]

**Before Ischemia**					**After Ischemia**				
**Con**	**Dbt**	**Dop**	**Dig**	**Lev**	**Con**	**Dbt**	**Dop**	**Dig**	**Lev**
Heart Rate (beats per minute)									
236 ± 3	350 ± 12*	298 ± 9*	259 ± 6	270 ± 5*	242 ± 8	350 ± 15^#^	298 ± 13^#^	269 ± 10	271 ± 7
Systolic LVP (mmHg)									
32 ± 1	73 ± 5*	55 ± 3*	49 ± 2*	51 ± 2*	29 ± 2	44 ± 3^#^†	40 ± 1^#^†	37 ± 2^#^†	35 ± 2^#^†
Diastolic LVP (mmHg)									
3 ± 1	2 ± 1	4 ± 1	6 ± 1	3 ± 2	13 ± 2†	13 ± 2†	13 ± 2†	14 ± 1†	18 ± 2†
Phasic (systolic-diastolic) LVP (mmHg)									
28 ± 2	71 ± 6*	52 ± 3*	43 ± 1*	48 ± 3*	16 ± 2†	32 ± 4^#^†	27 ± 2^#^†	23 ± 2^#^†	17 ± 3†
Systolic [Ca^2+^] (nM)									
258 ± 4	609 ± 48*	307 ± 14*	301 ± 10*	367 ± 31*	381 ± 27†	544 ± 56^#^	310 ± 19^#^	333 ± 22	369 ± 24
Diastolic [Ca^2+^] (nM)									
103 ± 2	140 ± 10*	108 ± 4	106 ± 2	110 ± 3	124 ± 9†	148 ± 13	107 ± 9	100 ± 6^#^	124 ± 10
Phasic (systolic-diastolic) [Ca^2+^] (nM)									
155 ± 5	469 ± 72*	198 ± 12*	194 ± 10*	257 ± 32*	257 ± 19†	396 ± 53^#^	204 ± 20	234 ± 17	245 ± 23

Values are expressed as mean ± SE. [CaCl_2_] was 1.25 mM. Con = Control, Dbt = Dobutamine, Dop = Dopamine, Dig = Digoxin, Lev = Levosimendan. Statistical significance was measured at *p < 0.05 vs. Control before ischemia, #p < 0.05 vs. Control after ischemia, †p < 0.05 after vs. before ischemia for all groups.

**Figure 3:**
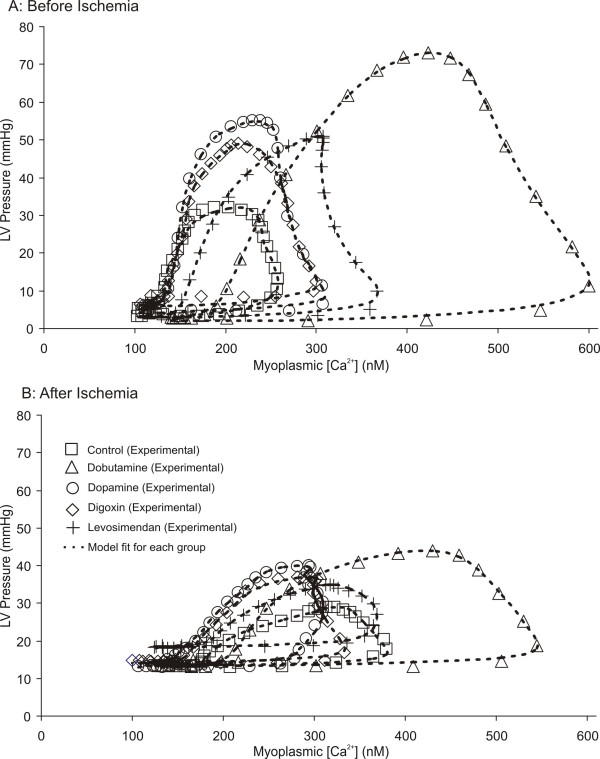
Typical plots of LVP and LVP_Mod _vs [Ca^2+^] averaged over all beats in the 2.5 second recordings for all groups before (A) and after (B) ischemia. Experimental data are represented by markers and the model fit is represented by dotted lines.

**Figure 4:**
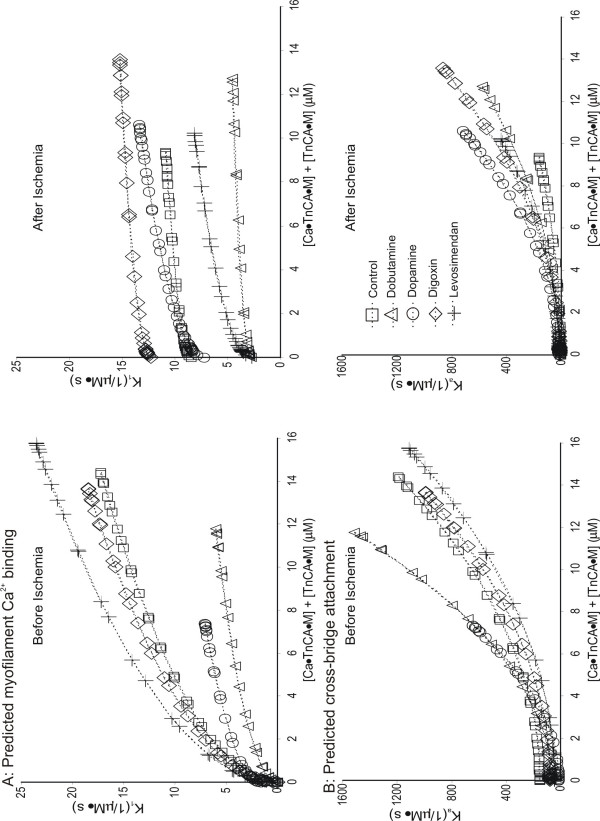
Model-predicted cooperative rate constants K_1 _(A) and K_a _(B) vs. the total number of formed cross-bridges, [Ca•TnCA•M] + [TnCA•M] for all experimental groups before (left) and after (right) ischemia. Ischemia alone resulted in a decrease in the maximum estimated number of formed cross-bridges from 14.2 μM to 9.2 μM. The maximal estimated number of formed cross-bridges with dopamine and dobutamine were less than control before ischemia (7.3 μM and 11.8 μM, respectively) and greater than control after (10.6 μM and 12.7 μM, respectively) ischemia. After ischemia digoxin increased the maximum estimated number of formed cross-bridges (13.6 μM) compared to control.

Experimentally measured LVP, plotted as a function of [Ca^2+^], is represented by symbols for all interventions before (A) and after (B) ischemia in figure 3. Corresponding LVP_Mod_, plotted as a function of [Ca^2+^], is represented by the dashed line for all interventions. Typical plots of the estimated cooperative parameters K_1 _(A) and K_a _(B) as a function of the total number of cross-bridges formed ([Ca•TnCA•M]+[TnCA•M]) for all groups before and after ischemia are shown in figure 4. Estimated model parameters before and after global ischemia is reported in table 3 and the effects of drugs and ischemia or model parameters are summarized in tables 4 and 5.

**Table 3:** Effects of positive inotropic drugs on model parameters when administered before and after global ischemia.

**Before Ischemia**					**After Ischemia**				
**Con**	**Dbt**	**Dop**	**Dig**	**Lev**	**Con**	**Dbt**	**Dop**	**Dig**	**Lev**
Second order rate constants					Second order rate constants				
*α*_*1 *_*(1/μM•s)*					*α*_*1 *_*(1/μM•s)*				
4.5 ± 0.3	2.2 ± 0.2*	2.3 ± 0.3*	4.2 ± 0.3	4.4 ± 0.5	0.5 ± 0.1†	0.5 ± 0.2†	1.2 ± 0.2^#^	1.1 ± 0.2^#^†	1.6 ± 0.4^#^†
*α*_*a *_*(1/μM•s)*					*α*_*a *_*(1/μM•s)*				
5.0 ± 0.2	8.0 ± 0.8*	14 ± 3*	5.8 ± 0.5	5.2 ± 0.3	1.7 ± 0.4†	4 ± 1^#^†	3.8 ± 0.6^#^†	4.2 ± 0.7^#^	2.4 ± 0.4†
*β*_*1 *_*(1/μM•s)*					*β*_1_*(1/μM•s)*				
0.12 ± 0.07	0.18 ± 0.15	2.2 ± 0.8*	0.2 ± 0.1	1.8 ± 0.9	10 ± 3†	4 ± 1^#^†	9.0 ± 0.5†	10 ± 2†	5.7 ± 2.0^#^†
*β*_*a *_*(1/μM•s)*					*β*_*a *_*(1/μM•s)*				
118 ± 12	83 ± 9	29 ± 13*	110 ± 10	56 ± 9*	5.2 ± 0.7†	10 ± 2^#^†	11 ± 3^#^†	13 ± 4^#^†	15 ± 8^#^†
*K*_*2 *_*(1/μM•s)*					*K*_*2*_*(1/μM•s)*				
418 ± 14	775 ± 82*	355 ± 68	485 ± 59	351 ± 62	151 ± 63†	275 ± 41†	262 ± 76	249 ± 28†	250 ± 9^#^†
First order rate constants					First order rate constants				
*K*_*3 *_*(1/s)*					*K*_*3 *_*(1/s)*				
429 ± 24	348 ± 46	174 ± 42*	434 ± 31	508 ± 63	271 ± 39†	270 ± 43	517 ± 26^#^†	480 ± 24^#^	178 ± 19^#^†
*K*_*4 *_*(1/s)*					*K*_*4 *_*(1/s)*				
18 ± 4	80 ± 17*	52 ± 9*	28 ± 9	37 ± 7*	60 ± 8†	90 ± 10^#^	65 ± 11	69 ± 6†	67 ± 13†
*K*_*d *_*(1/s)*					*K*_*d *_*(1/s)*				
484 ± 21	872 ± 29*	656 ± 54*	500 ± 20	593 ± 71	87 ± 13†	331 ± 27^#^†	183 ± 30^#^†	227 ± 77^#^†	325 ± 47^#^†
*K*_*d*_'*(1/s)*					*K*_*d*_'*(1/s)*				
765 ± 81	977 ± 32*	1218 ± 11*	728 ± 88	751 ± 64	377 ± 25†	375 ± 15†	378 ± 1†	450 ± 72†	398 ± 27†

Values are expressed as mean ± SE. Con = Control, Dbt = Dobutamine, Dop = Dopamine, Dig = Digoxin, Lev = Levosimendan. Statistical significance was measured at *p < 0.05 vs. Control before ischemia, #p < 0.05 vs. Control after ischemia, †p < 0.05 after vs. before ischemia in the presence or absence of drugs.

**Table 4:** Summary of drug-induced differences in model parameters compared to control.

	**Before Ischemia**				**After Ischemia**			
**Parameter**	**Dbt**	**Dop**	**Dig**	**Lev**	**Dbt**	**Dop**	**Dig**	**Lev**
*α*_*1 *_*(1/μM•s)*	↓	↓	NC	NC	NC	↑	↑	↑
*α*_*a *_*(1/μM•s)*	↑	↑	NC	NC	↑	↑	↑	NC
*β*_*1 *_*(1/μM•s)*	NC	↑	NC	NC	↓	NC	NC	↓
*β*_*a *_*(1/μM•s)*	NC	↓	NC	↓	↑	↑	↑	↑
*K*_*2 *_*(1/μM•s)*	↑	NC	NC	NC	NC	NC	NC	↑
*K*_*3 *_*(1/s)*	NC	↓	NC	NC	NC	↑	↑	↓
*K*_*4 *_*(1/s)*	↑	↑	NC	↑	↑	NC	NC	NC
*K*_*d *_*(1/s)*	↑	↑	NC	NC	↑	↑	↑	↑
*K*_*d*_' *(1/s)*	↑	↑	NC	NC	NC	NC	NC	NC

Arrows indicate directional differences in drug effects on model parameters from pre- and post-ischemic controls. NC = no change. Data obtained 25 min before and 35 min after global ischemia in the presence of drugs.

**Table 5:** Summary of differences in model parameters for each group after ischemia

**Parameter**	**Con**	**Dbt**	**Dop**	**Dig**	**Lev**
*α*_*1 *_*(1/μM•s)*	↓	↓	↓	↓	↓
*α*_*a *_*(1/μM•s)*	↓	↓	↓	NC	↓
*β*_*1 *_*(1/μM•s)*	↑	↑	↑	↑	↑
*β*_*a *_*(1/μM•s)*	↓	↓	↓	↓	↓
*K*_*2*_*(1/μM•s)*	↓	↓	NC	↓	↓
*K*_*3 *_*(1/s)*	↓	NC	↑	NC	↓
*K*_*4 *_*(1/s)*	↑	NC	NC	↑	↑
*K*_*d *_*(1/s)*	↓	↓	↓	↓	↓
*K*_*d*_' *(1/s)*	↓	↓	↓	↓	↓

Arrows indicate directional differences in model parameters after ischemia compared to before ischemia in each group. NC = no change. Data obtained 25 min before and 35 min after global ischemia in control hearts (no drug treatment).

### Effects of ischemia alone

The model described Ca^2+^-contraction coupling with relatively small errors between LVP_Mod _and LVP before (3.2 ± 0.5%) and after (2.2 ± 0.3%) IR injury. The model predicted:

a) Decreased cooperativity of Ca^2+ ^affinity for TnCA and cross-bridge association as demonstrated by the marked decreases in slopes of K_1 _and K_a _respectively, after ischemia.

b) Decreased cross-bridge dissociation rate constants K_d_, and K_d_' after ischemia.

c) Decreased K_2 _but increased K_4 _after ischemia; this indicates impaired myofilament Ca^2+ ^sensitivity when cross-bridges are attached.

### Effects of dopamine

The model described Ca^2+^-contraction coupling during dopamine administration with errors of 3.5 ± 0.7% and 2.6 ± 0.4% before and after ischemia, respectively. The model predicted:

a) Reduced interactions between Ca^2+ ^and TnCA before ischemia by dopamine as noted by the reduced values of α_1 _and K_3_, and by the increased value of K_4 _compared to control.

b) Increased interaction between Ca^2+ ^and TnCA after ischemia by dopamine as noted from the increased values of α_1 _and K_3 _compared to control.

c) Increased values of α_a_, K_d_, and K_d_' before ischemia by dopamine; this indicates improved cross-bridge cycling compared to control.

d) Improved cross-bridge kinetics compared to control after ischemia by dopamine as shown by increased estimates of α_a_, β_a_, and K_d_; however, this improvement was significantly less than the improvement noted with dopamine before ischemia.

### Effects of dobutamine

The model successfully described the relationship between [Ca^2+^] and LVP during dobutamine administration with low errors of 5.0 ± 0.5% before and 3.0 ± 0.4% after ischemia. The model predicted:

a) Reduced sensitivity of Ca^2+ ^binding to TnCA in dobutamine-treated hearts compared to control before ischemia as shown by the decrease in α_1_; however, Ca^2+ ^association and dissociation with actinomyosin cross-bridges, K_2 _and K_4 _respectively, was increased.

b) Marked increases in cross-bridge kinetics, i.e., α_a_, K_d_, and K_d_' before ischemia by dobutamine compared to control.

c) Reduced affinity of Ca^2+ ^for TnCA as assessed from decreased β_a _and increased K_4 _after ischemia by dobutamine compared to control.

d) Improved cross-bridge kinetics from increased α_a_, β_a_, and K_d _after ischemia by dobutamine compared to control.

e) Reduced cooperative myofilament Ca^2+ ^affinity (α_1_) and cross-bridge association (α_a_), increased basal rate constant of Ca^2+ ^binding to TnCA (β_1_), and reduced basal rate constant of cross-bridge association (β_a_) by dobutamine treatment after ischemia as compared to dobutamine treatment before ischemia. These predictions resulted in plots of K_1 _and K_a _(figure. 3) being flatter during administration of dobutamine after than before ischemia.

f) Reduced non-cooperative affinity of Ca^2+ ^for the formed cross-bridge, K_2_, and reduced cross-bridge dissociation rates K_d _and K_d_' by dobutamine treatment before ischemia as compared to dobutamine treatment after ischemia.

### Effects of digoxin

The model described Ca^2+^-contraction coupling during digoxin administration with 5.0 ± 0.6% and 2.0 ± 0.3% errors before and after ischemia, respectively. The model predicted:

a) No effects on myofilament Ca^2+ ^sensitivity or cross-bridge kinetics before ischemia by digoxin compared to control.

b) Increased sensitivity of Ca^2+ ^affinity for TnCA, α_1_, and dissociation of Ca^2+ ^from TnCA, K_3_, after ischemia by digoxin compared to control.

c) Improved cross-bridge cycling as noted from the increased values of α_a_, β_a_, and K_d _after ischemia by digoxin compared to control.

d) Reduced cooperative and non-cooperative affinities of Ca^2+ ^for TnCA, α_1 _and K_2 _respectively, by digoxin treatment after ischemia as compared to digoxin treatment before ischemia.

e) Enhanced Ca^2+ ^dissociation from cross-bridges, K_4_, by digoxin treatment after ischemia as compared to digoxin treatment before ischemia.

f) Reduced cross-bridge dissociations, K_d_, and K_d_', by digoxin treatment after ischemia compared to digoxin treatment before ischemia.

### Effects of levosimendan

The model described Ca^2+^-contraction coupling during administration of levosimendan with 4.0 ± 0.3% and 1.7 ± 0.3% errors before and after ischemia, respectively. The model predicted:

a) Depressed cross-bridge association with a decrease in β_a_, and enhanced myofilament Ca^2+ ^dissociation with an increase in K_4 _before ischemia by levosimendan compared to control; however, none of the other parameters were significantly changed by levosimendan compared to control before ischemia.

b) Increased sensitivity of Ca^2+ ^affinity for TnCA as noted from increased values of α_1 _and K_2_, and the decreased value of K_3 _after ischemia by levosimendan compared to control.

c) Enhanced basal rate constant of cross-bridge association, β_a_, and dissociation, K_d_, after ischemia by levosimendan compared to control.

d) Ischemia blunted the effects of levosimendan on all model parameters except the basal rate constant of Ca^2+ ^binding to TnCA, β_1_, and Ca^2+ ^dissociation from actinomyosin cross-bridges, K_4_.

## Discussion

The results indicate: 1) the four-state model with cooperativity is capable of interpreting changes in central and downstream regulation of contractility due to inotropic drugs in the presence and absence of IR injury in guinea pig isolated hearts from the phase-space relationship between [Ca^2+^] and LVP; 2) IR injury in the absence of inotropic agents resulted in reduced Ca^2+ ^affinity for TnCA, and decreased cross-bridge kinetics; 3) dopamine enhanced cross-bridge kinetics before and after ischemia; 4) dopamine decreased myofilament Ca^2+ ^affinity before ischemia but enhanced this affinity after ischemia; 5) in contrast, dobutamine reduced myofilament Ca^2+ ^sensitivity, and increased cross-bridge kinetics before and after ischemia; and 6) digoxin and levosimendan improved Ca^2+ ^affinity for TnCA and cross-bridge association after ischemia but neither drug substantially affected these parameters before ischemia.

### IR injury and Ca^2+^-contraction coupling

Theoretical models of Ca^2+^-contraction coupling during pharmacological and pathophysiological interventions have not been extensively examined. Winslow et al. [32] presented a computational model of excitation-contraction coupling during congestive heart failure in the guinea pig ventricular cell and demonstrated that simultaneous up-regulation of Na/Ca exchange and down-regulation of SR Ca-ATPase pump activity may account for the reduced amplitude of Ca^2+ ^transients in failing ventricular myocytes. Ch'en et al. [33] modeled cardiac metabolism during normal and ischemic conditions with particular attention to reperfusion arrhythmias resulting from sodium and calcium overload. However, neither of these models examined the effects of IR injury on Ca^2+^-mediated kinetic interactions between contractile and regulatory proteins.

Previously, we applied this same four-state model to study central and downstream mechanisms regulating Ca^2+^-contraction coupling in long-term cold IR injury compared to short-term warm IR injury [4]. Based on the model parameters, we predicted a marked decrease in actinomyosin cross-bridge cycling and reduced myofilament Ca^2+ ^sensitivity during hypothermic perfusion. The model also predicted better preservation of cooperativity in cross-bridge cycling and myofilament Ca^2+ ^handling after long term cold IR injury compared to short term warm IR injury despite cold-induced myoplasmic Ca^2+ ^loading.

The present investigation used dynamic changes in Ca^2+^-contraction coupling from experimentally derived phase-space diagrams of LVP vs. [Ca^2+^] that have been analyzed qualitatively [5], to examine changes in cross-bridge kinetics and myofilament Ca^2+ ^binding associated with IR injury. The model's results suggest that IR injury causes inefficient utilization of available [Ca^2+^] for contractile function. The model-estimated parameters revealed that IR injury results in reduced myofilament sensitivity for Ca^2+ ^binding; however, the basal rate constant of Ca^2+ ^binding to TnCA was increased. This may reflect excess myoplasmic Ca^2+ ^loading despite concomitant reduction of myofilament Ca^2+ ^sensitivity. IR injury also resulted in attenuation of model-predicted cross-bridge kinetics and formation of fewer cross-bridges. Decreased ATP production and hydrolysis may account for the reduction in cross-bridge dissociation rate constants, K_d _and K_d_'. These alterations in intracellular Ca^2+ ^homeostasis may have precipitated the rise in diastolic LVP and the reduction in LV compliance as noted from the phase-space diagram after ischemia in isolated hearts.

### Dopaminergic and β-adrenergic agonists and Ca^2+^-contraction coupling

Stimulation of dopaminergic and β_1_-adrenergic receptors by dopamine and dobutamine has been shown to increase myoplasmic cyclic adenosine 3', 5'-monophosphate (cAMP) levels and to activate cAMP-dependent protein kinase A. This protein kinase phosphorylates sarcolemmal Ca^2+ ^channels, SR Ca-ATPase, and troponin I on actin myofilaments [34,35]. However, it is unclear whether protein kinase-mediated phosphorylation of various proteins involved in contractile activation alters cross-bridge dynamics [9-11]. Results from the present model support our previous findings that dopamine reduces myofilament sensitivity to Ca^2+ ^before ischemia, but enhances it after IR injury [2,3,5]. Large increases in estimated rate constants of cross-bridge kinetics were also observed that may be attributed partially to the positive chronotropic effects of dopamine. Similarly, an increase in heart rate produced by dopamine may also contribute to the estimation of fewer formed cross-bridges before ischemia as observed in figure 4. After ischemia, dopamine resulted in a greater number of attached cross-bridges despite increased heart rate; this is likely associated with slower estimated cross-bridge dissociation rate constants (K_d _and K_d_'). Model parameters also suggested that dobutamine, like dopamine, decreased myofilament sensitivity to Ca^2+ ^before ischemia. In contrast to the findings with dopamine, myofilament Ca^2+ ^sensitivity was unchanged by dobutamine after ischemia. These differential effects of dopamine and dobutamine on myofilament Ca^2+ ^sensitivity after ischemia can also be observed from differences in phase-space morphology exhibited by the two drugs [5]. Cross-bridge kinetics were significantly increased by dobutamine before and after ischemia, and this was possibly related to an increase in heart rate. Our results show that whereas heart rates were increased approximately 50% by dobutamine prior to ischemia, the 1^st ^order rate constants (K_3_, K_d _and K_d_') increased by 400%, 80%, and 27% respectively. This discrepancy may be attributed to dobutamine-induced changes in myofilament Ca^2+ ^sensitivity and cross-bridge dissociation that are independent of dobutamine-induced increase in heart rate.

### Cardiac glycosides and Ca^2+^-contraction coupling

Digoxin is a clinically used Na^+^/K^+ ^ATPase inhibitor that may regulate the formation of actinomyosin cross-bridges [12], but its effects on Ca^2+ ^affinity for TnCA have yet to be completely quantified. Our model parameters suggested that digoxin did not alter myofilament Ca^2+ ^sensitivity or cross-bridge kinetics before ischemia. The model parameters suggest that digoxin may exert a positive inotropic effect simply due to the increase in myoplasmic [Ca^2+^] (upstream mechanism) in normal myocardium. In contrast, after ischemia digoxin was predicted to increase myofilament Ca^2+ ^sensitivity and cross-bridge kinetics, and to increase the estimated number of cross-bridges formed; however, no significant changes were seen in phase-space diagram size and shape from control after ischemia. This indicates that though the phase-space relationship between LVP and [Ca^2+^] appears unchanged by digoxin administration from control after IR injury [5], the model parameters revealed that the mechanism of Ca^2+^-contraction coupling had changed significantly from a primarily upstream mechanism before ischemia to a more central and downstream mechanism after ischemia. Another cardiac glycoside, oaubain, was shown to have similar effects on the ventricular myocardium of patients with heart failure as those we observed with digoxin after IR injury [13].

### Ca^2+ ^sensitizers and Ca^2+^-contraction coupling

A newer class of positive inotropic agents is the so-called myofilament Ca^2+ ^sensitizers. These drugs enhance contractility by either acting directly on TnCA to enhance the Ca^2+ ^binding mechanism (central mechanism), or by producing an increase in cross-bridge binding (downstream mechanism) in the absence of an increase in the total amount of activator Ca^2+ ^[36]. Levosimendan is a myofilament Ca^2+ ^sensitizer when administered at low concentrations, but also acts as a phosphodiesterase (PDE) inhibitor at higher concentrations [37]. Levosimendan is believed to bind to TnCA in the presence of Ca^2+ ^and to stabilize the Ca^2+^-TnCA complex without actually increasing Ca^2+ ^binding affinity with TnCA [15]. In guinea pig papillary muscles, paced at room temperature, levosimendan enhanced cross-bridge attachments while decelerating their detachments [16,17].

Our results from the estimated model parameters showed that levosimendan had little effect on myofilament Ca^2+ ^sensitivity or cross-bridge kinetics before ischemia. This lack of effect may be dose related or due to differences in experimental methods, specifically paced muscle strips vs. freely beating intact hearts. Our data suggests that levosimendan increases contractile function by increasing available [Ca^2+^] when administered before ischemia [5]. Levosimendan also increased heart rate before ischemia, but the model predicted unchanged values of K_d_, and K_d_'. This may result from a proportional increase in the number of cross-bridges and/or a prolonged attachment of cross-bridges. In contrast to its effects before ischemia, the model predicted that after ischemia levosimendan increases Ca^2+ ^affinity for TnCA and the basal rate constant of cross-bridge association to function more as a classic Ca^2+ ^sensitizing agent. But simultaneous increases in the predicted rate constants of dissociation of Ca^2+ ^from TnCA and cross-bridge dissociation may be related to PDE inhibition by this concentration of levosimendan after ischemia.

### Potential limitations

This model of Ca^2+^-contraction coupling relies on Ca^2+ ^transients obtained via an optic probe with a transmural measurement field and surface area of approximately 3 mm^2^. The model parameters are optimized to best fit isovolumic LVP, a more global measurement. One of the implicit assumptions of this model is that the local Ca^2+ ^transient is a true representation of Ca^2+ ^kinetics in the entire ventricular wall regardless of orientation of the individual myocytes which are known to be markedly different from endocardium to epicardium [38].

Because these hearts were unpaced, changes in heart rate with inotropic interventions likely confound some of the conclusions drawn from our model parameters. However, only by allowing hearts to beat at their inherent rhythm can we fully understand the physiologic impact of these drugs on the intact heart. Controlling the heart rate would lead to changes in the peak values and kinetics of the measured Ca^2+ ^transients and thus result in LVP values that are non-physiologic.

Another potential limitation of this model is the presence of lumped parameters as a result of oversimplification of the mechanism of protein-protein interactions. For example, cross-bridge dissociation is modeled as one rate constant (K_d _or K_d_') and so reactions associated with myosin ATPase activity are not distinguished. As a result the model parameters represent relative, and not absolute, rates of myofilament Ca^2+ ^sensitivity and cross-bridge kinetics.

## Conclusion

In summary, we used a 4-state computational model to predict the relationship between [Ca^2+^] and LVP during the cardiac cycle in the guinea pig isolated heart when exposed to positive inotropic drugs before and after global ischemia. The changes in estimated values of rate constants before and after ischemia appeared to coincide with known changes in cross-bridge formation and myofilament Ca^2+ ^sensitivity and helped to resolve controversial findings regarding drug effects on central and downstream mechanisms after ischemia. The model also predicted differential changes in contractile protein interactions based on post-synaptic receptor adrenergic or dopaminergic activation. Moreover, the model parameters predicted that digoxin and levosimendan have little effect on central and downstream mechanisms prior to ischemia but that both significantly improve myofilament Ca^2+ ^sensitivity and cross-bridge cycling after ischemia. These mathematical characterizations should facilitate future studies that aim to analyze the effects of preconditioning or heart failure on contractile kinetics. The phase-space relationship between experimentally measured [Ca^2+^] and LVP is useful as a framework to model the theoretical effects of these interventions on Ca^2+^-contraction coupling.
